# Development of a Cell-Based Bioassay for Phospholipase A^2^-Triggered Liposomal Drug Release

**DOI:** 10.1371/journal.pone.0125508

**Published:** 2015-05-06

**Authors:** Ahmad Arouri, Jakub Trojnar, Steffen Schmidt, Anders H. Hansen, Jan Mollenhauer, Ole G. Mouritsen

**Affiliations:** 1 MEMPHYS-Center for Biomembrane Physics, Department of Physics, Chemistry, and Pharmacy, University of Southern Denmark, Odense, Denmark; 2 Lundbeckfonden Center of Excellence NanoCAN, Institute for Molecular Medicine, University of Southern Denmark, Odense, Denmark; 3 Molecular Oncology Group, Institute for Molecular Medicine, University of Southern Denmark, Odense, Denmark; Okayama University, JAPAN

## Abstract

The feasibility of exploiting secretory phospholipase A_2_ (sPLA_2_) enzymes, which are overexpressed in tumors, to activate drug release from liposomes precisely at the tumor site has been demonstrated before. Although the efficacy of the developed formulations was evaluated using *in vitro* and *in vivo* models, the pattern of sPLA_2_-assisted drug release is unknown due to the lack of a suitable bio-relevant model. We report here on the development of a novel bioluminescence living-cell-based luciferase assay for the monitoring of sPLA_2_-triggered release of luciferin from liposomes. To this end, we engineered breast cancer cells to produce both luciferase and sPLA_2_ enzymes, where the latter is secreted to the extracellular medium. We report on setting up a robust and reproducible bioassay for testing sPLA_2_-sensitive, luciferin remote-loaded liposomal formulations, using 1,2-distearoyl-*sn*-glycero-3-phosphatidylcholine/1,2-distearoyl-*sn*-glycero-3-phosphatidylglycerol (DSPC/DSPG) 7:3 and DSPC/DSPG/cholesterol 4:3:3 as initial test systems. Upon their addition to the cells, the liposomes were degraded almost instantaneously by sPLA_2_ releasing the encapsulated luciferin, which provided readout from the luciferase-expressing cells. Cholesterol enhanced the integrity of the formulation without affecting its susceptibility to sPLA_2_. PEGylation of the liposomes only moderately broadened the release profile of luciferin. The provided bioassay represents a useful tool for monitoring active drug release *in situ* in real time as well as for testing and optimizing of sPLA_2_-sensitive lipid formulations. In addition, the bioassay will pave the way for future in-depth *in vitro* and *in vivo* studies.

## Introduction

The potential use of liposomes as a drug carrier in anticancer treatment was first highlighted by Gregoriadis et al. in the early seventies [1]. Twenty years later, Doxil received a market clearance from the US FDA as the first anticancer liposomal formulation, which opened the door for several other anticancer lipid-based medicinal products [2,3]. Despite the fact that liposomes can enhance the physico-chemical properties and pharmacokinetics of the encapsulated drugs as well as reduce their systemic toxicities, passive drug release from liposomes is often associated with sub-optimal drug efficacy [2]. This urged the need in the past decade to develop new approaches for active and selective drug delivery as well as for triggered drug release at the target site [4]. One promising strategy are bio-responsive drug carriers, whose characteristics can be altered in response to the specific microenvironment of the cancer or to external stimuli [5].

Secretory phospholipase A_2_ (sPLA_2_) is a lipolytic enzyme that catalyzes the cleavage of phospholipids at position *sn*-2 [6,7]. Due to the suspected role of sPLA_2_ in tumorigenesis and metastasis, some subtypes of the enzyme (e.g. sPLA_2_ IIA) are overexpressed in several cancer types, like prostate, breast and colon cancer [8–12]. Depending on the cancer type, cancer stage, and presence of metastasis, 28–100% of the cancer patients can have aberrant levels of sPLA_2_ that are in average 6–8 times higher in the tumor compartment than in serum [13–15]. This phenomenon opened new avenues for designing sPLA_2_-sensitive lipidic systems for active and selective drug delivery, and thereby helped to overcome some of the limitations of conventional liposomes. After their accumulation in cancer tissues overexpressing sPLA_2_ [16], the liposomes will be enzymatically degraded releasing their payload precisely at the target. The feasibility of this platform has been demonstrated before for doxorubicin- and cisplatin-loaded liposomes [17,18], liposome-forming lipid-like anticancer prodrugs [5,19,20], and double lipid-prodrug systems [21,22]. It has also been postulated that the locally generated hydrolytic products, i.e. fatty acids and lysolipids, due to their membrane perturbing properties, can enhance cellular drug uptake [23]. The cytotoxicity and tumor growth inhibition of the formulations were evaluated in *in vitro* and *in vivo* studies, respectively [17], nevertheless, the sPLA_2_-assisted drug release profile could not be determined because of the absence of an appropriate bioassay.

Bioluminescence assays are highly sensitive and easy to handle, and therefore are widely used as a detection strategy in medical and biological research [24–27]. In a complex ATP-driven oxidative decarboxylation reaction, the luciferin substrate, for example, is converted by luciferase enzyme to oxyluciferin emitting yellow-green light as a byproduct. Depending on the luciferase system and reactants concentrations, the bioluminescent flashes can vary in kinetics, duration and intensity [28,29]. The use of living cells in the bioassay is not inferior to the use of lysate or pure enzyme, although the generated signal is generally weaker [30]. Also, the amphiphilic and relatively small luciferin molecule can freely cross lipid membranes when exists in the unionized form. This property allows the remote-loading of luciferin, which is a weak acid with pK_a_ around 2.8 [31], into liposomes as well as the free diffusion of luciferin through cell membranes. The ionization of luciferin inside the liposomes prevents the back diffusion of luciferin to the bulk [27].

In order to enable analyses of drug release profiles with living cells, we devised a system with luciferin remote-loaded sPLA_2_-sensitive liposomes and MCF-7 breast cancer cells engineered to produce firefly luciferase (*luc2* gene) and sPLA_2_ enzymes, so that drug release can be monitored by emergence of luminescence. The bioassay thus resembles release and uptake of a drug with membrane-crossing potential. We tested the bioassay using free luciferin as well as two lipid formulations composed of the uncharged DSPC (1,2-distearoyl-*sn*-glycero-3-phosphatidylcholine) and the anionic DSPG (1,2-distearoyl-*sn*-glycero-3-phosphatidylglycerol) without and with cholesterol, namely DSPC/DSPG 7:3 molar ratio (denoted PCPG) and DSPC/DSPG/cholesterol 4:3:3 molar ratio (denoted PCPGch). Moreover, the effect of PEGylation on the luminescence profile was determined. The bioassay worked reliable and allowed to determine that cholesterol enhanced the integrity of the formulation without affecting its susceptibility to sPLA_2_, while PEGylation moderately broadened the release profile of luciferin.

## Materials and Methods

### Materials

1,2-Distearoyl-*sn*-glycero-3-phosphocholine (DSPC), 1,2-distearoyl-*sn*-glycero-3-phosphatidylglycerol (DSPG), and cholesterol were purchased from Corden Pharma LLC (Switzerland). 1,2-Dipalmitoyl-*sn*-glycero-3-phosphoethanolamine-N-[methoxy(polyethylene glycol)-2000] (DPPE-PEG 2000) was purchased from Avanti Polar Lipids (Alabaster, AL, USA). D-Luciferin (sodium salt) was purchased from Regis Technologies, Inc. (USA). All other chemicals and solvents were obtained from Sigma-Aldrich Chemicals Co. (Germany). All substances were used as received without any further purification or modification. If not otherwise specified, the concentration of the substances was calculated from the weight of the dry materials (weight/volume).

### Stable cell lines

MCF-7 cells were obtained from ATCC. Stable cell lines were generated by using a modified Flp-In system (Invitrogen), cloning the open reading frames (orfs) of the luciferase 2 (*luc2*; DQ904455) and the human *PLA2G2A* genes (NM_001161729) into an expression plasmid under the control of the constitutive Cytomegalovirus (CMV) promoter. Cells were cultivated in DMEM medium (Sigma—Aldrich) supplemented with 10% fetal bovine serum (Sigma—Aldrich), 1% pen-strep (Sigma—Aldrich) and 10 μg/ml insulin (Sigma–Aldrich) in a humidified atmosphere at 37°C and with 5% CO_2_. To confirm expression of *PLA2G2A*, total RNA was purified from cells using the RNeasy Plus kit (Qiagen) and treated with the DNAse I mix (Roche) as recommended. RNA quantity and purity was determined using a NanoDrop ND-1000 spectrophotometer (Saveen Werner). Reverse transcription was carried using the RevertAid H Minus First Strand cDNA Synthesis Kit (Fermentas) with 1 μg of total RNA preparation in 20 μl as recommended by the manufacturer’s instructions. Quantitative RT-PCR (qRT-PCR) reactions were performed in triplicate wells using a *PLA2G2A*-specific TaqMan assay (Life Technologies; Hs00179898_m1). Human *ACTB* and *GAPDH* Endogenous control assays (Life Technologies) served as references for normalization. The reactions were performed on a StepOnePlus machine (Life Technologies) using a cycling profile of 50°C for 2 minutes and 95°C for 15 minutes followed by 40 cycles of 95°C for 15 seconds and 60°C for 1 minute. The cycle thresholds (CT) of the specific targets were normalized to *GAPDH* and *ACTB* and the relative quantitative evaluation was performed using the Biogazelle program. To determine the *luc2* activity per cell, 7,500 cells per well were seeded in 7-plicate into 96-well culture plates. After 24 hours, 100 μl of the Steady-Glo® reagent (Promega) were added to each well and the plates were incubated at 37°C for 25 minutes. The luminescence detection was performed using the Wallac VICTOR^3^ TM 1420 Multilabel Counter. In parallel, 7,500 cells per well were seeded in 7-plicate, and 24 hours later, 20 μl of CellTiter-Blue® (Promega) was added to each well and the plates were incubated at 37°C for 3 hours. The readout was performed in the Wallac VICTOR^3^ TM 1420 Multilabel Counter (PerkinElmer) at the wavelength of 560_Ex_/590_Em_. Luminescence was divided by the cell viability readout to obtain the relative luc2 activity per cell and the values obtained for MCF-7 cells with luc2 only inserted were set to 1.0 to serve as the reference point.

### Preparation of lipid vesicles

The lipid mixtures were prepared in chloroform/methanol 4:1 (v/v), after which the organic solvents were evaporated under vacuum. The formed lipid films were hydrated in an aqueous magnesium acetate buffer (120 mM, pH 6.0) with the aid of vortexing to get a final concentration of 20 mM. If necessary, the pH of the lipid-containing solution was re-adjusted to 6.0. The lipid suspension was freeze-thawed 10 times before being extruded 15 times through two 100 nm polycarbonate filters using an Avanti Mini Extruder (Avanti Polar Lipids, Alabaster, AL, USA) at a temperature higher than the phase transition temperature of the lipid mixture. PEGylated liposomes were prepared by post-insertion via the mixing of the luciferin-loaded liposomes with DPPE-PEG 2000 solution at room temperature (24°C) for one hour [32]. The size and polydispersity of the liposomal preparations were controlled with dynamic light scattering (DLS) (Brookhaven-BI-200SM goniometer).

### Remote loading of luciferin into preformed liposomes

The freshly extruded liposomes (20 mM, 2–4 ml) in 120 mM magnesium acetate buffer (pH 6.0) were dialyzed to exchange buffer in 2 x 1 liter of 120 mM potassium sulfate buffer (pH 6.0) under gentle stirring at 6–8°C for 2 x 24 hours using dialysis membranes (Spectra/Por Float-A-Lyzer G2, MWCO 3.5–5 kD) from Spectrum® Laboratories, Inc. The osmolarity of the potassium sulfate buffer was adjusted to match the osmolarity of the magnesium acetate buffer using D(+)glucose solution. After exchanging buffer, the liposomes were added to a luciferin solution in 120 mM potassium sulfate buffer (pH 6.0) to achieve a final luciferin-to-lipid weight ratio of 1:4, and the mixture was kept under gentle shaking for 48 hours (protected from light) at room temperature (24°C) to allow the remote loading of luciferin using the established acetate/acetic acid gradient. Untrapped (free) luciferin was removed by dialysis in 2 x 1 liter of 120 mM potassium sulfate buffer (pH 6.0) under gentle stirring at 6–8°C for 2 x 24 hours. The amount of encapsulated luciferin was determined by lysing the liposomes using Triton X-100, diluting the luciferin solution using 0.5 M potassium carbonate buffer (pH 11.5), and measuring luciferin absorbance at 385 nm using a NanoDrop UV/Vis spectrophotometer (ND-1000, Thermo Scientific). Luciferin concentration was calculated using an extinction coefficient of 18,200 M^-1^ cm^-1^ [33]. Triton X-100 showed no effect on luciferin absorbance (unpublished observations). The phospholipid concentration was determined using a procedure adapted from Bartlett’s phosphate assay [34].

### Luminescence bioassay

For the bioassay, 2,500 cells per well in 100 μl DMEM medium with supplements were seeded in a sterile white 96-well plate. For the free luciferin assay the cells were incubated for 24 hours before use, whereas for testing the liposomes and to allow for accumulation of sufficient sPLA_2_ enzyme the cells were incubated for 48–72 hours before the test. The experiments were performed in duplicates or more, and the luminescence signal from the luciferase-luciferin reaction was recorded in a FLUOstar Omega Microplate Reader (BMG LAB- TECH) thermostated at 37°C. After the equilibration of the solutions and the microplate seeded with the living MCF-7 cells at 37°C for few minutes, the bioassay was started by the automated injection via machine pumps of free luciferin or luciferin-loaded liposomes (intact or lysed) to the cells. Luciferin and Triton X-100 solutions were prepared in 120 mM potassium sulfate buffer (pH 6.0).

## Results and Discussion

### Engineering of luc2/sPLA_2_-positive and control cell lines

**Fig 1:**
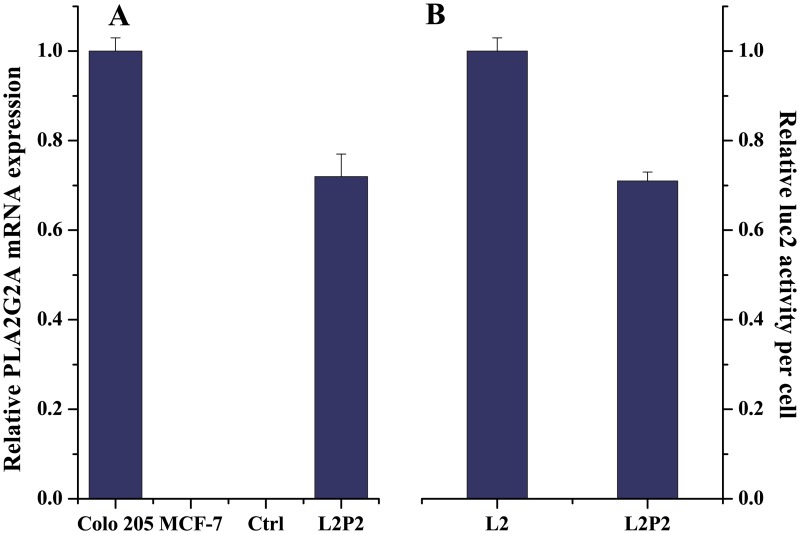
Analyses of stable cell lines. (A) Determination of relative *PLA2G2A* mRNA levels in L2P2 and control cells, normalized to GAPDH and ACTB levels. Values are referred to Colo 205 cells with high endogenous *PLA2G2A* mRNA levels [35]. (B) Relative luciferase activity of L2 and L2P2 cells normalized to cell numbers as determined by a cell viability assay as substitute readout. Error bars represent standard error of the mean (SEM).

To design a cellular test system, we selected MCF-7 breast cancer cells for stable insertion of the open reading frames for human *PLA2G2A* and the *luc2* reporter gene under the control of the constitutive CMV promoter, referred to as L2P2 cells. MCF-7 cells with an empty expression plasmid inserted were used as negative control (Ctrl). Correspondingly, engineered cells were first monitored by qRT-PCR for *PLA2G2A* expression. In comparison to colorectal cancer Colo 205 cells, known to express high sPLA_2_ levels [35], non-modified parental MCF-7 and Ctrl cells showed virtually undetectable *PLA2G2A* mRNA expression, whereas L2P2 cells approached the endogenous levels of Colo 205 cells (see Fig 1A).

We next engineered MCF-7 cells with stable insertion of *luc2* only, referred to as L2 cells, which—based on absence of *PLA2G2A* expression—would serve as negative control for unspecific release. L2 and L2P2 cells where analyzed in endpoint measurements for their luciferase activity per cell via normalization to an independent cell viability assay performed in parallel. The assay indicated that, while the luciferase activity was comparable, L2P2 cells had slightly less (0.71 ± 0.02—fold) activity compared to L2 cells (see Fig 1B), which is likely due to simultaneous high level *PLA2G2A* expression in L2P2 cells, which competes for available transcription factors.

### Bioassay establishment

**Fig 2:**
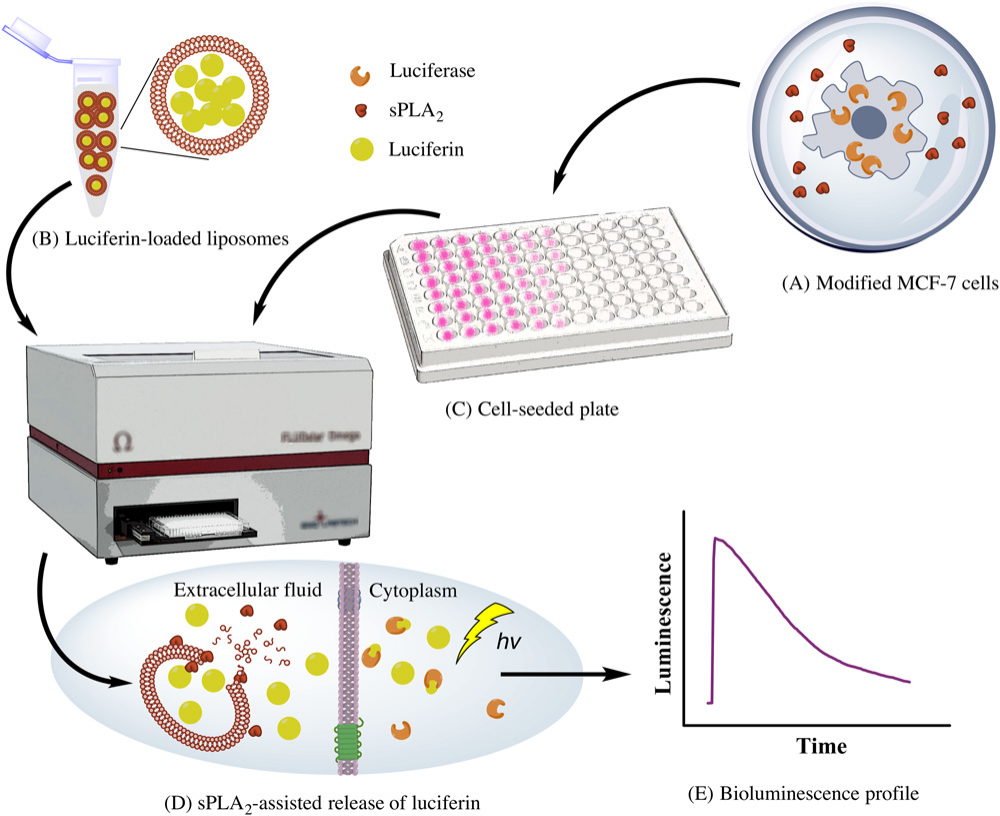
Schematic illustration of the principle of the developed firefly luciferase bioassay for following *in situ* sPLA_2_-assisted release of luciferin from liposomes. (A) Engineering of a stable MCF-7 breast cancer cell line to produce firefly luciferase and sPLA_2_ enzymes. The sPLA_2_ enzyme will be secreted to the extracellular fluid. (B) Preparation of luciferin remote-loaded liposomal formulations. (C) Plating of the engineered MCF-7 cell line, and incubation of the seeded plate for 48–72 hours. (D) The sPLA_2_-assisted hydrolysis of liposomes and the release of the encapsulated luciferin. The liberated luciferin can freely translocate across the cell membrane, and will be oxidized quickly by the intracellular luciferase enzyme in a light-emitting reaction. (E) Typical readout of the bioluminescence signal from the release assay.

The bioassay is based on the rationale that the luciferase enzyme will remain in the cytoplasm, whereas the sPLA_2_ enzyme expressed by L2P2 cells will be secreted to the extracellular compartment. The strategy of the bioassay is depicted in Fig 2. Upon the addition of luciferin-loaded liposomes to L2P2 cells, the liposomes will be degraded by sPLA_2_ releasing the encapsulated luciferin. Exogenous luciferin can freely enter the cells, and it will be rapidly oxidized by luciferase in a light-emitting reaction. By contrast, no signal would be expected from L2 cells that lack *PLA2G2A* expression. The luminescence profile shown in Fig 2 follows the flash kinetics with a fast increase in signal followed by a fast decay, which can be attributed to the accumulation of inhibitory products [28,29]. Expectedly, the kinetics of the luminescence profiles generated using living cells is much slower than the kinetics observed with the pure enzyme system [29,36,37].

**Fig 3:**
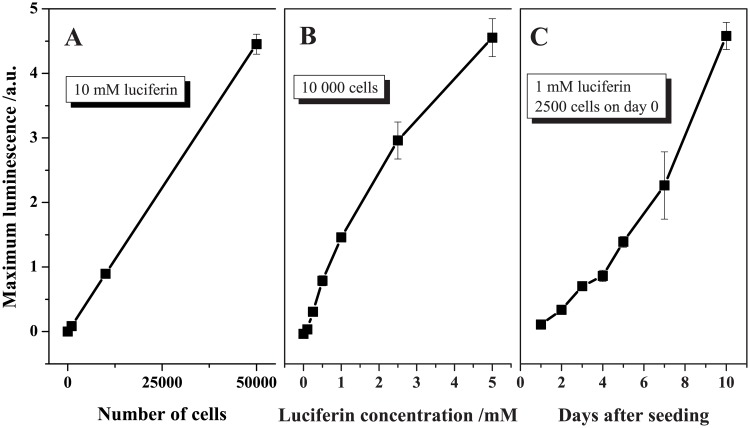
Testing of the bioassay using free luciferin. Dependence of the maximum of the luminescence profile generated upon the addition of luciferin to L2 cells on (A) number of seeded cells (0–50,000 L2 cells, 10 mM luciferin), (B) luciferin concentration (0–5 mM luciferin, 10,000 L2 cells), and (C) number of days after the seeding of 2,500 L2 cells (1–10 days, 1 mM luciferin). Lines are inserted only as a guide to the eye. Error bars represent the standard deviation (SD).

**Fig 4:**
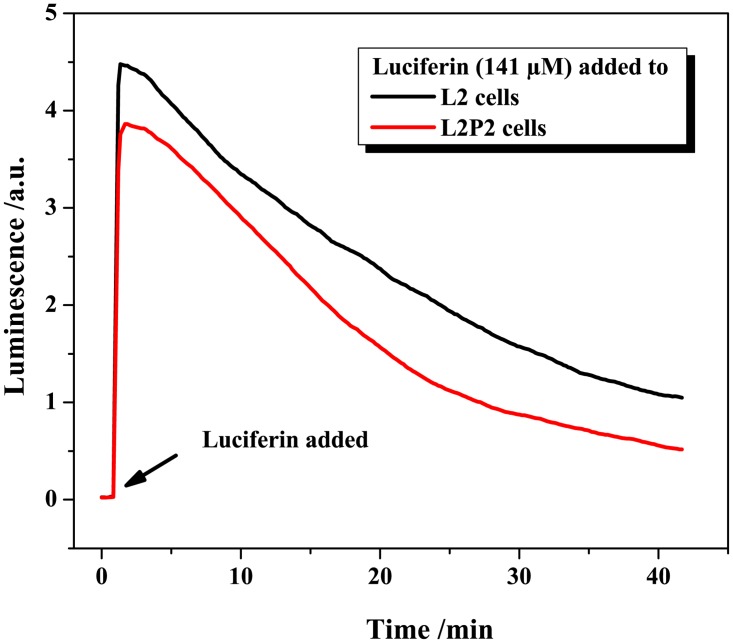
Testing of L2 and L2P2 cells. Luminescence profiles generated upon the addition of 141 μM luciferin to L2 and L2P2 cells.

The bioassay was first tested using free luciferin and L2 cells. As shown in Fig 3A, the (maximum) luminescence signal is proportional to the number of seeded cells. The luciferase-luciferin reaction follows a hyperbolic-Michaelis-Menten-like kinetics with increasing luciferin concentration (Fig 3B), which is comparable to what has been reported before with the pure enzyme [38]. The correlation between the signal and the number of days after cell seeding (see Fig 3C) is apparently exponential, which agrees well with the anticipated exponential growth of mammalian cells in cell cultures [39]. A comparison between the luminescence profiles recorded upon the addition of 141 μM luciferin to L2P2 and L2 cells is presented in Fig 4 and confirms that L2P2 cells produce slightly less luciferase than L2 cells.

### Utilization of the bioassay for testing liposomal formulations

The new cell-based bioassay was finally applied to test two lipid formulations composed of the uncharged DSPC and the anionic DSPG at 7:3 molar ratio (denoted PCPG) and DSPC/DSPG with cholesterol at 4:3:3 molar ratio (denoted PCPGch). The lipid composition was chosen based on earlier experiments on the enzymatic activity of human sPLA_2_ subtype IIA on liposomes [17]. In general, and due to the cationic nature of the enzyme, human sPLA_2_ requires a certain threshold (around 30%) of anionic lipids for activity [40]. The incorporation of cholesterol should improve the integrity and stability of the liposomes without significantly affecting the susceptibility of the liposomes to human sPLA_2_ [41–43].

The number-weighted average diameter of the prepared liposomes was 90 ± 25 nm (polydispersity < 0.1). As demonstrated in an earlier work at our lab on the colloidal stability of luciferin-loaded phosphatidylcholine/phosphatidylglycerol (PC/PG) formulations, the buffer exchange and the remote-loading processes did not drastically alter the size and polydispersity of the liposomes [44]. The remote-loaded formulations were found to be highly stable for about two months when stored at 6–8°C, and the colloidal stability of the preparations was further enhanced by PEGylation [44]. The liposomes were remote-loaded with luciferin using the established acetate/acetic acid gradient [27]. Before testing the luciferin-loaded lipid formulations PCPG and PCPGch, their luciferin and lipid content was analyzed. PCPG liposomes contained 33 ± 18 μg luciferin per mg lipid, whereas PCPGch liposomes had increased amounts (52 ± 17 μg luciferin per mg lipid) of remote-loaded luciferin. Giving the initially high luciferin concentration used in the remote-loading process (luciferin to lipid ratio of 25 weight%), the encapsulation efficiency of luciferin, which represents the percentage of the total amount of luciferin that was remote-loaded, was relatively low, i.e. 9 ± 3% for PCPG and 16 ± 2% for PCPGch. It should be noted that the luciferin-to-lipid ratio in the final formulation is not controllable, and therefore it was only possible to fix one of the concentrations when comparing the different formulations.

**Fig 5:**
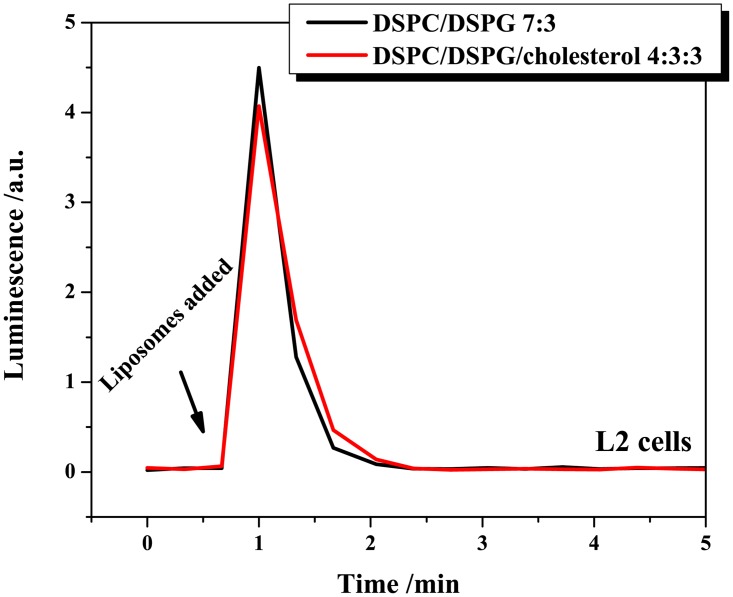
Testing of lysed luciferin-loaded liposomes. Luminescence profiles generated upon the addition of luciferin-loaded liposomes lysed with 0.5 wt.% Triton X-100 to L2 cells. Two formulations were tested (100 nm diameter liposomes), DSPC/DSPG 7:3 (PCPG, 117 μM luciferin and 880 μM lipid) and DSPC/DSPG/cholesterol 4:3:3 (PCPGch, 74 μM luciferin and 1220 μM lipid).

To confirm the encapsulation of luciferin, the bioassay was performed for liposomes lysed with Triton X-100 surfactant using L2 cell line. As shown in Fig 5, the luminescence signal was comparable for both formulations, however, in contrast to free luciferin (see Fig 4), the signal generated from lysed liposomes only lasted for a short time (about one minute). This is probably due to the high cytotoxicity of Triton X-100 [45].

**Fig 6:**
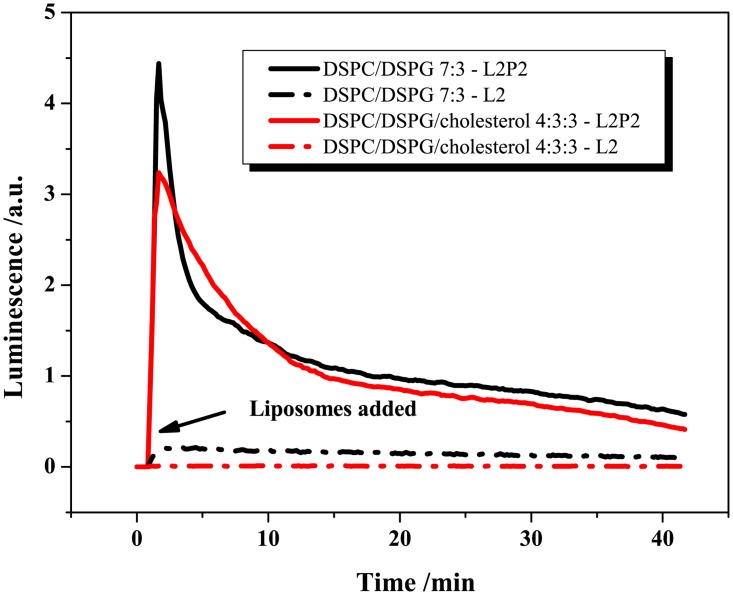
Testing of luciferin-loaded liposomes. Luminescence profiles generated upon the addition of luciferin-loaded liposomes to L2P2 and L2 cells. Two formulations were tested (100 nm diameter liposomes), DSPC/DSPG 7:3 (PCPG, 33 μM luciferin and 833 μM lipid) and DSPC/DSPG/cholesterol 4:3:3 (PCPGch, 33 μM luciferin and 265 μM lipid).

The luminescence profiles determined for both formulations (see Fig 6) demonstrate that L2P2 can robustly release the luciferin payload from PCPG (33 μM luciferin and 833 μM lipid) and PCPGch (33 μM luciferin and 265 μM lipid) liposomes. Despite the different lipid concentration used (fixed luciferin concentration), the profile of both formulations was highly comparable. However, PCPGch, which displayed slightly higher loading efficacy (Fig 5), showed a reduced peak release and a slightly delayed kinetics (Fig 6). In the negative control assay with L2 cells, low luminescence signal was observed with the cholesterol-free formulation indicating modest luciferin leakiness, whereas the inclusion of cholesterol reduced the leakiness of the liposomes. The products of the enzymatic hydrolysis, i.e. fatty acids and lysolipids, are cytotoxic in themselves in micromolar concentrations [23]. Although the decay in the signal after the maximum luminescence was linked to the accumulation of inhibitory products from the luciferase reaction [28,29], it cannot be ruled out that the decay was partially due to the toxic effects of the liberated fatty acids and lysolipids.

### Effect of PEGylation

**Fig 7:**
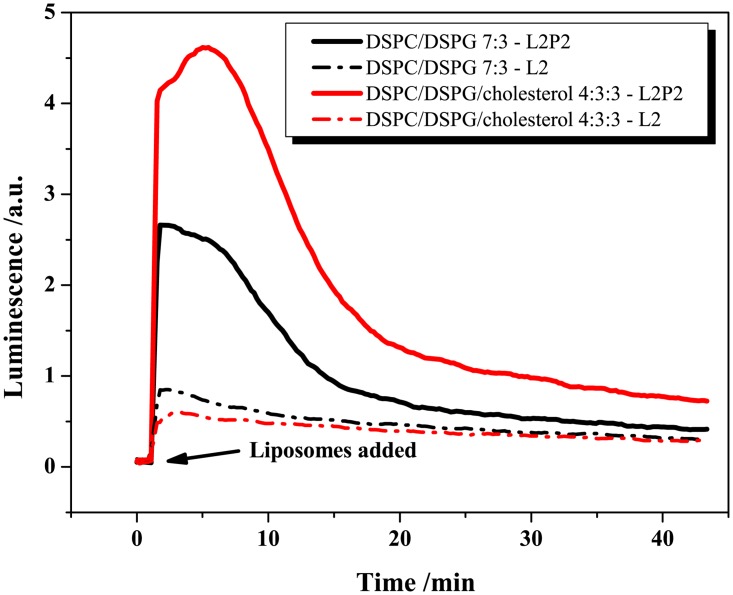
Testing of the effect of PEGylation. Luminescence profiles generated upon the addition of PEGylated luciferin-loaded liposomes to L2P2 and L2 cells. The lipopolymer DPPE-PEG 2000 (5 mol%) was added via post insertion to the loaded liposomes. Two formulations were tested (100 nm diameter liposomes), DSPC/DSPG 7:3 with (PCPG, 33 μM luciferin and 833 μM lipid) and DSPC/DSPG/cholesterol 4:3:3 (PCPGch, 107 μM luciferin and 833 μM lipid).

The lipopolymer DPPE-PEG 2000 (1,2-dipalmitoyl-*sn*-glycero-3-phosphoethanolamine-N-poly(ethylene glycol)-2000) was added to the luciferin-loaded PCPG and PCPGch liposomes via the post-insertion method [32]. From earlier studies, the PEGylation is expected to boost the enzymatic activity and accelerate the hydrolysis of the liposomes [22,41,46,47]. The effect of PEGylation with 5 mol% of DPPE-PEG 2000 on the stability and sPLA_2_-susceptibility of the liposomes is presented in Fig 7, where in this case the lipid concentration was fixed to 833 μM (excluding the lipopolymer). As the results show, the PEGylation counteracted the stabilizing effect of cholesterol and increased the leakiness of both formulations. In addition, the PEGylation broadened the luciferin release profile in the assay on L2P2 cells. The higher area under the curve of the profile of PCPGch was due to the higher luciferin concentration used in test (107 μM compared with 33 μM).

It appears from the luminescence profiles reported in this paper, which extend over 40 minutes, that luciferin release is a continuous process. This concurs with earlier observations that the openings formed in the lipid membrane during the hydrolysis tend to reseal quickly due to the fast diffusional rearrangement of the membrane components [48,49].

## Conclusions

The recent years have witnessed an increasing interest in the design of smart and multi-purpose vehicles for active and selective drug delivery [4]. Exploiting sPLA_2_ enzymes for triggering drug release precisely at the target site, where the tumor resides, is a promising approach furnished by its applicability *in vivo* as well as by the large body of data already available on the activity of sPLA_2_ enzymes. However, the vast majority of the data were collected from studies on model systems and computer simulations [5,6]. Therefore, the effects of the complex and dynamic cancer microenvironment on the liposome physico-chemical properties and on the enzymatic activity of sPLA_2_ are so far not well understood. The efficacy of anticancer sPLA_2_-susceptible liposomes was measured earlier using cytotoxicity assays *in vitro* and tumor growth inhibition *in vivo* in a mouse xenograft model [17]. Yet, there are no data on the pattern of sPLA_2_-assisted drug release *in vitro* or *in vivo* due to the lack of a proper bio-relevant model for testing the platform. In addition, it is remains challenging to achieve a fine balance between high drug encapsulation efficiency, liposome stability, and formulation sensitivity to sPLA_2_. From our initial testing, it appears that DSPC/DSPG 7:3 formulation is a favorable substrate for sPLA_2_, which at the same time can provide high drug entrapment efficiency. The inclusion of cholesterol can enhance the formulation stability and drug retention without substantially altering the drug release profile. Interestingly, the PEGylation of the liposomes can be used to modulate the kinetics of lipid hydrolysis and drug release on the expense of perturbing the membrane permeability barrier of the liposomes. We believe the newly developed bioassay described in the present paper will not only allow the monitoring of active drug release *in situ* in real time, but it will also prove useful for testing and optimization of sPLA_2_-sensitive lipid formulations. Furthermore, the bioassay will aid our understanding of the behavior of sPLA_2_-triggered drug release in future *in vitro* and *in vivo* studies.
